# B Cell Dynamics and Transitional B Cells in Long COVID

**DOI:** 10.3390/cimb47040245

**Published:** 2025-04-01

**Authors:** Zoia R. Korobova, Natalia A. Arsentieva, Natalia E. Liubimova, Oleg K. Batsunov, Anastasia A. Butenko, Albina E. Kokoeva, Natalia G. Kucherenko, Victor A. Kashchenko, Ekaterina V. Boeva, Anna O. Norka, Anastasia A. Knizhnikova, Vadim V. Rassokhin, Nikolay A. Belyakov, Areg A. Totolian

**Affiliations:** 1 Laboratory of Molecular Immunology, Saint Petersburg Pasteur Institute, ul. Mira, 14, 197101 Saint Petersburg, Russia; zoia-korobova@yandex.ru (Z.R.K.); batsunov@gmail.com (O.K.B.); aabutenko15@gmail.com (A.A.B.); kathrine.boeva@gmail.com (E.V.B.); norka-anna@mail.ru (A.O.N.); nasya.sur@yandex.ru (A.A.K.); ras-doc@mail.ru (V.V.R.); beliakov.akad.spb@yandex.ru (N.A.B.); totolian@spbraaci.ru (A.A.T.); 2 Department of Immunology, First Pavlov State Medical University of St. Petersburg, Leo Tolstoy str, b. 6-8, 197022 Saint Petersburg, Russia; 3 The Federal State Budgetary Institution ‘North-Western District Scientific and Clinical Center Named after L.G. Sokolov Federal Medical and Biological Agency’, Prospekt Kul’tury, 4, 194291 Saint Petersburg, Russia; albina080194@mail.ru (A.E.K.); nataliadoc@mail.ru (N.G.K.); surg122@yandex.ru (V.A.K.)

**Keywords:** long COVID, cytokines, B cells, transitional B cells, CD27, CD38, Th2

## Abstract

Background: Long COVID is characterized by persistent symptoms following acute SARS-CoV-2 infection. This study aims to evaluate immune system markers, including antigen-specific antibodies, B cell subsets, and Th2-related cytokines, in individuals with long COVID and to investigate their potential impact on the development of this condition. Methods: We analyzed blood plasma from 63 individuals diagnosed with long COVID based on clinical presentation and 47 healthy individuals with COVID-19 history but no clinical symptoms. Antigen-specific IgG antibodies were measured using commercial ELISA kits. Lymphocyte subpopulations were assessed via flow cytometry and a gating strategy based on CD27 and CD38. Th2 cytokines (IL-4, IL-5, IL-13) were quantified using the xMAP multiplex assay. Results: We noted no significant differences in IgG levels between groups. Notably, individuals with long COVID demonstrated a higher percentage of naive mature B cells (CD27−CD38+), while transitional (CD27−CD38+++) and double-negative (DN, CD27−CD38-) cells were significantly reduced. Elevated levels of IL-5 and IL-13 were observed in long COVID patients. Classification analysis revealed that the percentage of transitional B cells (CD27−CD38+++) was a strong predictor of long COVID. Conclusions: Our findings highlight alterations in B cell dynamics among individuals with long COVID, which may contribute to autoimmune processes.

## 1. Introduction

The World Health Organization (WHO) defines the post-COVID-19 condition, or long COVID, as a wide variety of long-lasting symptoms within three months after COVID-19 [1].

Long COVID, a condition that persists in many individuals following the initial COVID-19 infection, mostly persists within the first two years [2]. However, it manifests in a diverse array of symptoms. Among the most common are fatigue, cognitive dysfunction, often referred to as “brain fog”, and respiratory difficulties. Emerging evidence suggests that these manifestations may be linked to immune dysregulation, prompting a closer examination of the immune system’s role in this complex condition [3].

The connection between systemic responses and local immunity in neural tissues underscores the importance of studying immunity in relation to neuroinflammatory alterations and cognitive impairments in long COVID. Additionally, it is crucial to explore how these immune responses correlate with other symptoms like shortness of breath, fatigue, and increased susceptibility to viral infections.

While T-cellular responses are known for their precision in targeting viral infections, the routine assessment of long-lasting immunity against COVID-19 still heavily relies on the detection of antigen-specific antibodies [4]. It is important to note that while antibodies are unable to cross the blood–brain barrier, cytokines can [5], highlighting the importance of cytokine-producing cells and minor subpopulations of lymphocytes in our investigation. Therefore, it is worth evaluating not only the antibody-producing cells but also the smaller populations within the B-cellular branch of lymphocytes to better understand their contributions to immunity and potential implications for long COVID development.

By exploring these components, we hope to uncover valuable insights into the complex interplay of immune responses in the context of COVID-19 and its long-term effects.

## 2. Materials and Methods

This study was performed in medical facilities of the Saint Petersburg Pasteur Institute, Pavlov First Saint Petersburg State Medical University, and the North-Western Scientific and Clinical Center named after L.G. Sokolov. This study was approved by the Ethical Committee of the Saint Petersburg Pasteur Institute (protocol #84, 16 February 2023). All participants included in this study signed written consent forms.

This study included 63 individuals with long COVID. Diagnosis was based on persistent psychoneurological (i.e., cognitive dysfunction based on the Montreal Cognitive Assessment, anxiety and depression based on the Hospital Anxiety and Depression Scale) and somatic symptoms (fatigue and dyspnea associated with COVID-19, lasting for >12 weeks). Other criteria for study inclusion were age (18–60 years old) and the fact of previously diagnosed COVID-19 proven via PCR. The median age was 38 years (29–48), with mostly women present in this study (79%, *n* = 50, vs. 21%, *n =* 13). In 85% of cases, previous COVID-19 was characterized as mild, whereas in 15% it was moderate or severe. In 82% of cases studied, subjects had COVID-19 more than once. Only 41% were previously vaccinated against COVID-19.

The most common symptoms in the acute phase of COVID-19 included fever (>38 °C), shortness of breath, lowered blood oxygen saturation, and confusion (Table 1).

When administered for long COVID diagnosis and treatment, patients most commonly noted that their quality of life was lowered due to hair loss (57.1%, *n =* 36), bad appetite (52.4%, *n =* 32), unspecified stomach pain (46%, *n =* 29), and fluctuating blood pressure (100%, *n =* 63).

Most patients (74.6%) had other chronic diseases of non-infectious origin in remission: gastrointestinal disorders (31.7%, *n =* 20), cardiovascular diseases (6.3%, *n =* 4), and urinary and renal system disorders (9.5%, *n =* 6). In 15 individuals, body mass index was above 25 kg/m^2^.

Due to the non-specificity of long COVID as a disease and the heterogeneity of the studied group, we recommend that the readers be mindful of the results’ interpretation.

As controls, we invited 47 healthy individuals with no somatic or psychoneurological dysfunction associated with COVID-19 or long COVID. The median age was 39 (35–45) years, with mostly women present in this study (70%, *n =* 33 vs. 30%, *n =* 24).

All experiments were performed within less than 6 h after blood collection. Peripheral blood samples were collected into vacuum test tubes added with K3-EDTA anticoagulant (followed by processing for analyzing the relative and absolute counts of major T and B cell subsets with multicolor flow cytometry).

To assess antigen-specific antibodies in blood plasma, we used a commercial ELISA kit to assess total specific IgG against the SARS-CoV-2 N protein, designed and manufactured by the Saint Petersburg Pasteur Institute (registered for commercial use with the Federal Service for Surveillance in Healthcare (Roszdravnadzor, date of registration 14 February 2021)). Another commercial ELISA kit for detecting IgG against the SARS-CoV-2 RBD was manufactured by LabPac (Saint Petersburg, Russia).

To assess lymphocyte subpopulations in peripheral blood, we used flow cytometry. B cell whole peripheral blood samples were stained with the following anti-human monoclonal antibodies: CD38-PE (Beckman Coulter, Brea, CA, USA), CD5-ECD (Beckman Coulter, Brea, CA, USA), CD27−PC7 (Beckman Coulter, Brea, CA, USA), CD19-APC/Cy7 (BioLegend, Inc., San Diego, CA, USA), and CD45-Krome Orange (Beckman Coulter, Brea, CA, USA). As a basis for the gating strategy, we assessed CD27 and CD38 co-expression among CD45 + CD19 + CD5- cells [6].

Summarized data on B cell phenotyping and functional activity of subpopulations within this study are presented in Table 2.

To assess the levels of cytokines required for B cell activation and maturation, we used the xMAP (Luminex, Austin, TX, USA) multiplex assay. For this study, we measured concentrations of G-CSF, GM-CSF, IFN-γ, IL-1β, IL-2, IL-4, IL-5, IL-6, IL-7, IL-8, IL-10, IL-12 (p70), IL-13, IL-17A, MCP-1 (MCAF), MIP-1β, and TNF-α with a Bio-Rad cytokine multiplex assessment kit (Bio-Rad, Hercules, CA, USA). For statistical analysis and data visualization, we used GraphPad Prism 8 (Dotmatics Inc., Boston, MA, USA), SPSS Statistics 23 (IBM, Armonk, NY, USA), and Microsoft Excel 2016 (Microsoft, Redmond, WA, USA) software. This study was performed at the cytometry and biomarkers core facility center at the Saint Petersburg Pasteur Institute, Russia.

Statistical analysis included Spearman’s rank-order correlation between clinical presentation of long COVID and clinical course of COVID-19, the Mann–Whitney U-test for pairwise comparisons, and QUEST classification analysis.

## 3. Results

Based on clinical presentation, we noted associations between blood pressure fluctuation and severity of COVID-19 (Spearman’s Rho = 0.45, *p* = 0.02). Other markers of acute COVID-19 did not seem to affect the clinical presentation of long COVID.

**Figure 1:**
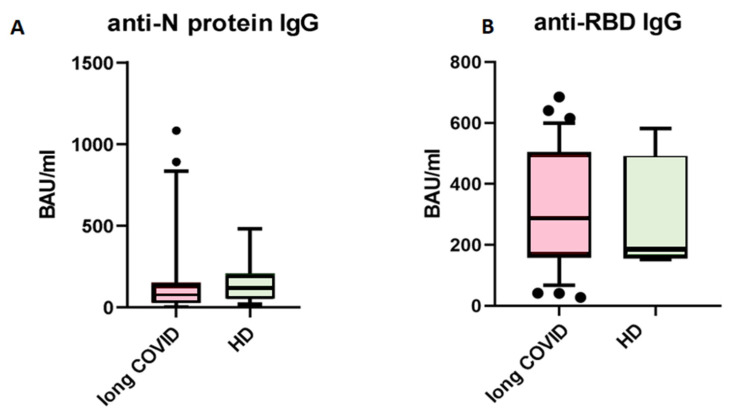
Comparison of circulating IgG antibodies in blood plasma of patients with long COVID (*n =* 63) and healthy donors with no symptoms of long COVID (*n =* 47). (**A**) anti-N protein IgG; (**B**) anti-RBD IgG. No statistical differences were found via Mann–Whitney U-test. The Y axis presents BAU/mL, the central line stands for median, and the whiskers are at 10–90 percentiles.

First, we analyzed levels of circulating IgG antibodies against the N-protein of SARS-CoV-2. When comparing patients with long COVID and healthy donors with no symptoms after COVID-19, we saw no statistical differences between groups (Figure 1). The cut-off value for this kit is set at 120 BAU/mL, indicating that results above this level are interpreted as ‘positive’, suggesting effective immunity against SARS-CoV-2 infection.

**Figure 2:**
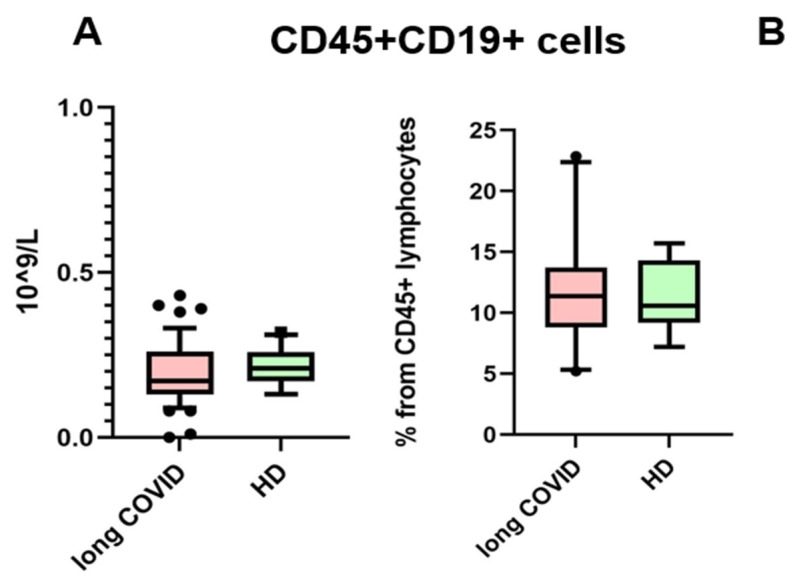
Comparison of CD45 + CD19+ lymphocytes in the whole blood of patients with long COVID and healthy donors (HDs). (**A**) Comparison of absolute counts (cells × 10^9^/L); (**B**) comparison of percentages (from CD45% lymphocytes). No statistical differences were found via Mann–Whitney U-test. Box represents median and Q25–Q75. Bars stand for the 10–90 percentile.

The presence of anti-SARS-CoV-2 N-protein antibodies in both cohorts is unsurprising, due to the fact that IgG antibodies provide immunological memory for more than 365 days, only changing in subclass prevalence [12]. It is important to note that we specifically selected a kit for detecting anti-N-protein antibodies in order to identify individuals who had COVID-19 rather than those who were merely vaccinated, since most vaccines in the Russian Federation are linked to the production of anti-RBD S-protein antibodies [13]. In our study, we observed that the levels of IgG antibodies against the N-protein of SARS-CoV-2 did not show significant differences between the long COVID group and the control group. Therefore, IgGs against the N-protein are unlikely to be a contributing factor in the observed B cell characteristics between these groups. For the next step, we compared the absolute count and percentage of CD45+ CD19+ cells. No statistically significant differences were noted between groups. The results are presented in Figure 2.

**Figure 3:**
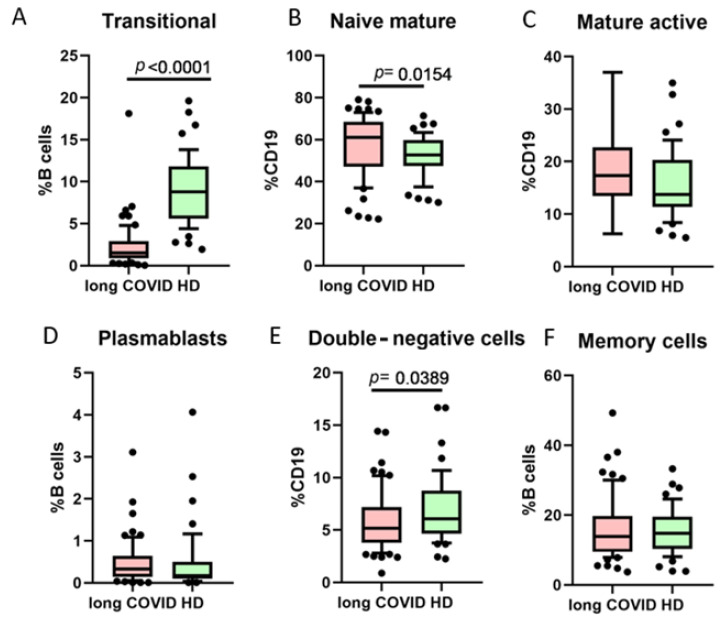
Comparison of B cell minor subsets based on CD27/CD38 co-expression in long COVID patients and healthy donors (HDs). (**A**) Transitional cells (CD27−CD38++), (**B**) naive mature cells (CD27−CD38+), (**C**) mature activated (CD27+CD38+), (**D**) plasmablasts (CD27+++CD38++), (**E**) double-negative (CD27−CD38−), (**F**) memory cells (CD27+CD38−). Box represents median and Q25–Q75. Bars stand for the 10–90 percentile.

One of the B cell classifications is based on their functional activity and co-expression of markers CD27 and CD38 (Figure 3). We noted that long COVID patients had a statistically significant higher percentage of naive mature cells (CD27−CD38+), whereas transitional cells (CD27−CD38+++) and double-negative (DN, CD27−CD38−) cells were lower when compared to healthy donors.

**Figure 4:**
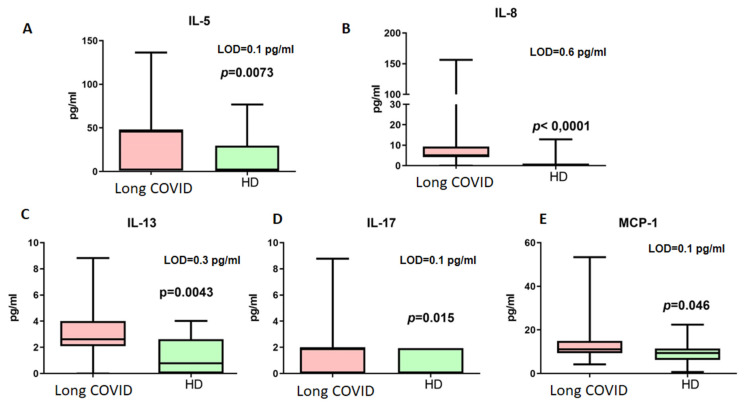
Cytokine concentrations (pg/mL) in blood plasma of long COVID patients (Long COVID) and healthy donors (HDs). Only cytokines demonstrating statistically significant differences are presented ((**A**) IL-5, (**B**) IL-8, (**C**) IL-13, (**D**) IL-17, (**E**) MCP-1). LOD—limit of minimal detection according to manufacturers’ manual. Box represents median and Q25–Q75. Bars stand for min and max values.

The next step included measurements of cytokines associated with immune responses in long COVID. The results are presented in Figure 4.

Among the detected cytokines, only IL-4, IL-5, and IL-13 are associated with Th2-dependent immunity. For further analysis of B cellular responses, we addressed their concentrations. Thus, it should be noted that IL-4 did not demonstrate statistically significant differences in concentrations between patients with long COVID (median 0.29 pg/mL vs. 0.19 pg/mL, *p* > 0.05, LOD = 0.1 pg/mL).

**Figure 5:**
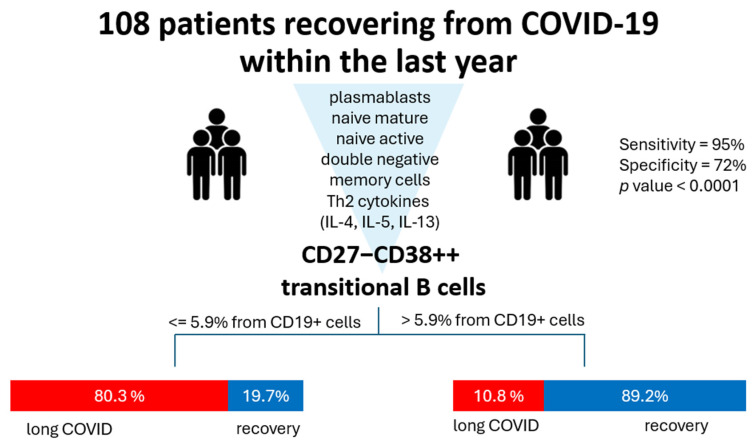
QUEST classification analysis for long COVID (group 1, red) and healthy donors (group 2, blue). Parameters included in the analysis were percentages of plasmablasts, naive mature, naive active, double-negative (CD27−CD38-), and transitional cells and levels of Th2-associated cytokines (IL-4, IL-5, IL-13). The independent variable with the highest impact is CD27−CD38+++ transitional cell percentage with a cutoff value ≥ 5.94. For validation, we used cross-validation, number of folds: 10. The *p* value for this classification tree was <0.0001. Specificity for such a model is 72%, whereas sensitivity is 95%.

Finally, we analyzed all the abovementioned markers in terms of classification analysis to determine what has the most prominent impact on the development of long COVID symptoms. For that, we used a QUEST decision tree analysis, which demonstrated that out of all the assessed parameters, the percentage of cells with phenotype CD27−CD38++ (transitional) is effective in terms of post-COVID prognosis (Figure 5). In 83.3% of cases, CD27−CD38+++ ≥ 5.94% allowed the model to classify the patients into a ‘long COVID’ group.

Additional data on the studied data are presented in Table 3.

## 4. Discussion

Whereas most patients with long COVID often present with lymphopenia [14], it is rarely seen as a deficiency in B cells. Usually, the B cell population levels do not differ from the control in the recovery period [15]. We noted the same tendency in our study: although overall levels of CD19+ cells did not differ from controls, patients with long COVID demonstrated an altered distribution of smaller subpopulations. Phetsouphanh et al. noted the same in their study, where changes in subpopulations are present in individuals at different time points since the infection. In the same study, general B cell levels did not differ between groups [16].

The study by Golovkin et al. demonstrates that patients with COVID-19 exhibit lower levels of B cells compared to healthy donors. Although CD19+ cells in the study are linked to the severity of the disease, this relationship is atypical, i.e., patients with severe COVID-19 had B cell counts that were comparable to those of healthy individuals [17]. The review article by Mansourabadi raises an intriguing point regarding the role of B cells in acute COVID-19. Their findings suggest that there is not a deficiency of B cells during this phase of the illness; rather, the issue lies in the dysregulation of antibody formation. This dysregulation is attributed to a decreased number of follicular T helper (Th) cells, which impairs the formation of germinal centers. As a result, B cells experience extrafollicular activation, leading to the production of antibodies characterized by low levels of somatic hypermutation and an increased secretion of proinflammatory cytokines [18]. However, our previous study demonstrated no statistical differences in Tfh levels in acute COVID-19 [19], unlike B cells that witnessed an imbalance in distribution between subpopulations.

In our previous research, we investigated the role of B cells in the context of COVID-19, focusing on the B cell receptor genetic recombination [20]. This process results in the formation of unique DNA rings known as KREC molecules. Alongside T cell receptor excision circles (TRECs), KREC molecules serve as biomarkers for defining errors in immune responses. Our previous findings indicate that KREC molecules and thus, B cells and their receptors, play a significant role in determining COVID-19 outcomes and severity mediated via the development of immune deficiencies [21]. This highlights the critical importance of B cell assessment in understanding the immune landscape during COVID-19, highlighting the need for further exploration of B cell dynamics in infectious diseases.

The study by Berger et al. explores T cells and B cells in long COVID [15]. There, an increase in transitional cells is described in the first 200 days since COVID-19 in patients with long COVID, which contradicts our findings. Such discrepancy can be possibly explained by the fact that in Berger’s study this subpopulation was detected via a different phenotyping strategy (IgA/M+CD38+++). Transitional B cells can be categorized into several developmental subsets. Among these are T1 B cells, which have a high expression of IgM, and T2 B cells, which express both IgM and IgD. These B cells go through various tolerance checkpoints, where those that strongly bind to self-antigens are eliminated. In contrast, cells with intermediate or low affinity for self-antigens, as well as those that do not recognize self at all, survive and circulate for approximately three weeks to search for their target antigens. Transitional B cells can differentiate into either marginal zone (MZ) B cells or follicular B cells. MZ B cells sample antigens and can expand independent of T cell assistance when they encounter their specific antigens [22]. Although we did not perform additional analysis to differentiate transitional B cells based on their subtype (T1, T2, or T3), it is still worth mentioning that usually in autoimmune diseases, the percentage of type 3 B cells is reduced, while CD27+ transitional B cells are increased compared to healthy controls [23].

An increase in naive mature cells provides antigen-specific protection, but a lack of transitional cells leads to an imbalance in autoimmunity regulation. In vitro studies suggest regulatory and tolerance-inducing roles for transitional B cells [24]. Transitional cell levels in the peripheral blood are reported to be altered in people with autoimmune conditions such as multiple sclerosis, neuromyelitis optica spectrum disorders, systemic lupus erythematosus, Sjögren’s syndrome, rheumatoid arthritis, systemic sclerosis, and juvenile dermatomyositis [7]. As post-COVID-19 is often associated with neurological symptoms, the role of these cells in neuroinflammation is intriguing. These cells have regulatory functions, but the effects of their dysfunction differ among autoimmune diseases, including those followed by neurocognitive dysfunction (multiple sclerosis, neuromyelitis, etc.). For example, in multiple sclerosis, a lower percentage of these cells marks higher activity of an autoimmune lesion on MRI [25]. We noted that statistically these cells have a high impact on long COVID development. The nature of long COVID is still a question of debate, but one of the often-discussed points is whether autoimmune processes can kickstart the symptom development [26,27].

It must be added that while our study focused on immune markers and autonomic and cognitive dysfunction, we acknowledge that the clinical course of acute COVID-19 plays a critical role in shaping long-term outcomes. Existing data highlight that acute disease severity consistently predicts long COVID manifestations [28]. Notably, vaccination status may modulate this risk, as vaccinated individuals exhibit milder long COVID trajectories, even when adjusting for acute severity [29]. Long COVID transcends somatic pathology, intersecting with socioeconomic and psychological determinants. Lower socioeconomic status and pre-existing mental health conditions are linked to prolonged symptom burden, possibly due to chronic stress [30].

## 5. Conclusions

Long COVID is a heterogeneous condition that emerges as a continuation of SARS-CoV-2 infection, manifesting in diverse phenotypes and affecting multiple organ systems. In this study, we focused on immune dysregulation in long COVID, particularly Th2-mediated responses and B cell dynamics, which may contribute to its complex pathophysiology. Our findings underscore the urgent need to establish standardized diagnostic criteria for long COVID, and we propose transitional B cells as a potential biomarker for identifying immune imbalance in affected patients.

However, long COVID is not solely an immunological disorder. Its trajectory is shaped by COVID-19 severity, vaccination status, and biopsychosocial factors, requiring a multidisciplinary approach.

## Figures and Tables

**Table 1:** Main characteristics of COVID-19 in the acute phase preceding long COVID.

Symptom	Number of Participants Presenting the Symptom	% of the Studied Cohort
Fever (>38 °C)	53	84.1
Shortness of breath	25	39.6
Lowered blood oxygen saturation (95% and less)	4	6.3
Lowered blood pressure below 90/60 mm Hg	6	9.5%
Ventilatory support in the intensive care unit	3	4.7

**Table 2:** Phenotypes and function of B cellular subsets based on CD27 vs. CD38 co-expression.

CD27 vs. CD38 Phenotype	Function
CD27−CD38++	Transitional B cells, immature bone marrow-derived regulatory subpopulation [7]
CD27−CD38+	Mature naive cells in secondary lymphoid organs, initiate rapid antigen-associated B cell responses and B cell receptor diversification [8].
CD27+CD38+	Mature activated cells, early IgM memory [9].
CD27+++CD38++	Plasmablasts, responsible for long-term B-cellular immunity [10].
CD27+CD38−	Resting memory B cells, providing memory reservoir [9].
CD27−CD38−	Mature antigen-experienced B cells with an expression profile of developmental markers [11].

**Table 3:** Values included in QUEST classification analysis.

Value	Long COVID (Median, Q25–Q75)	Recovery (Median, Q25–Q75)
B lymphocytes (% from CD45+ cells)	13.91 (9.72–14.43)	11.21 (8.37–14.91)
Transitional cells (% from B lymphocytes)	2 (0.19–3.4)	9.52 (6.11–12.34)
Naive mature cells (% from B lymphocytes)	61.16 (47.13–68.47)	56.32 (49.12–59.78)
Mature active cells (% from B lymphocytes)	17.31 (13.42–22.73)	12.32 (11.41–24.3)
Plasmablast cells (% from B lymphocytes)	0.33 (0.14–1.04)	0.21 (0–1.3)
Memory B cells (% from B lymphocytes)	13.88 (9.49–30.77)	14.02 (10.12–25.81)
DN cells (% from B lymphocytes)	5.15 (3.79–7.18)	5.5 (5.15–8.44)
IL-4 (pg/mL)	0.29 (0.1–1.47)	0.29 (0.1–0.58)
IL-5 (pg/mL)	46.88 (1.12–46.88)	1.12 (1.12–29.57)
IL-13 (pg/mL)	2.62 (2.1–4.01)	0.78 (0–2.61)

## Data Availability

Data available on request due to ethical restrictions.
